# Lens Connexin Channels Show Differential Permeability to Signaling Molecules

**DOI:** 10.3390/ijms21186943

**Published:** 2020-09-22

**Authors:** Peter R. Brink, Virginijus Valiunas, Thomas W. White

**Affiliations:** Department of Physiology and Biophysics, Stony Brook University School of Medicine, Stony Brook, New York, NY 11794-8661, USA; peter.brink@stonybrook.edu (P.R.B.); virginijus.valiunas@stonybrook.edu (V.V.)

**Keywords:** connexin, gap junction, channel, permeability, second messenger, lens

## Abstract

Gap junction channels mediate the direct intercellular passage of small ions as well as larger solutes such as second messengers. A family of proteins called connexins make up the subunits of gap junction channels in chordate animals. Each individual connexin forms channels that exhibit distinct permeability to molecules that influence cellular signaling, such as calcium ions, cyclic nucleotides, or inositol phosphates. In this review, we examine the permeability of connexin channels containing Cx43, Cx46, and Cx50 to signaling molecules and attempt to relate the observed differences in permeability to possible in vivo consequences that were revealed by studies of transgenic animals where these connexin genes have been manipulated. Taken together, these data suggest that differences in the permeability of individual connexin channels to larger solutes like 3′,5′-cyclic adenosine monophosphate (cAMP) and inositol 1,4,5-trisphosphate (IP_3_) could play a role in regulating epithelial cell division, differentiation, and homeostasis in organs like the ocular lens.

## 1. Introduction

Tissues require continuous exchange of information between their constituent cells to coordinate activities required for growth and development. This communication is mediated in part through the activation of intracellular signal transduction by extracellular growth factors to generate second messengers, which can then be directly propagated between adjacent cells through the connexin channels present in gap junctions. Connexin channels link the cytoplasm of adjacent cells and allow for the direct exchange of ions, metabolites, and second messengers [1]. Different connexin proteins form channels that are functionally distinct in terms of their conductance and permeability to small molecules [2,3,4,5,6]. Genetic studies in mice have suggested that the functional differences observed between connexins in vitro are important in vivo, since the loss of one isoform cannot be compensated for by replacement with another connexin within the same cell type or tissue [7,8,9,10,11]. This raises the intriguing prospect that differences in the permeability to second messengers is one possible reason for why so many different connexin genes are required in any given cell type [5]. 

The permeability of gap junction channels to cAMP was documented before the first connexin genes were even cloned. Experiments where rodent ovarian granulosa cells and myocardial cells were placed in co-culture showed that these heterologous cell types could communicate through gap junction channels. Both types of cell responded to cell-specific hormones through cyclic AMP-dependent mechanisms, and exposure of the co-cultures to a hormone specific for one cell type caused the cAMP-dependent response in the other cell type in a cell contact-dependent fashion [12]. This study was the first to suggest that cAMP could permeate gap junction channels and could initiate physiological responses in neighboring cells. The permeation of gap junction channels by IP_3_ was initially observed between rat hepatocytes during the same time in which the first connexin genes were cloned from the liver [13,14]. The authors directly injected IP_3_ into isolated pairs or small clusters of cells and used the Ca^2+^ dye fura-2 to detect Ca^2+^ release in neighboring hepatocytes triggered by the passage of IP_3_ through gap junction channels [14]. This work provided the first evidence that the second messenger IP_3_ could be transmitted between cells through gap junction channels.

Many studies have shown that the intercellular communication provided by gap junctional communication and growth factor signaling pathways is important for the normal growth of vertebrate lens [15,16,17,18,19,20,21,22]. Gap junctional communication in the lens has been proven to be required for normal cell proliferation, differentiation, and metabolic coordination [23,24,25,26]. The lens is an ideal system for exploration of differential permeabilities of gap junctions to second messengers, as it abundantly expresses three major connexins in two types of cells. Cx43 and Cx50 are present in lens epithelial cells, while Cx46 and Cx50 form the gap junction channels between lens fibers [25,27]. In addition, there is an established literature on the consequences of genetic manipulation of lens connexins in mice [24,25,28] that can be directly correlated with any second messenger permeation data. The functional properties of lens connexin channels have been thoroughly studied in a variety of expression systems [29,30,31,32,33,34,35]. Recently, Cx43, Cx46, and Cx50 have been shown to have markedly different permeabilities to second messenger molecules that influence cellular signaling [36,37]. Here, we review the published evidence on differences in second messenger permeability through connexin channels made of Cx43, Cx46, and Cx50 and speculate on how this may contribute to the regulation of the intercellular communication necessary for normal growth of the lens.

## 2. Lens Connexin Permeability

### 2.1. Cx50 Has Significantly Reduced Permeability to cAMP Compared to Cx43 and Cx46

Differences in cAMP permeability through gap junction channels formed by different connexins have been measured using two different approaches: a quantitative patch clamp assay relying on activation of the cyclic nucleotide-gated channel SpIH in cell pairs [3,36,38,39] or the microscopic observation of the cell-to-cell passage of fluorescent ε-cAMP [36]. Using either approach, Cx50 displayed significantly reduced cAMP permeability compared to both Cx43 and Cx46. The cAMP permeability of Cx46, although much higher than Cx50, was still significantly lower when compared to that of Cx43. Calculation of the solute flux rate gave values of 6095, 1220, and 176 cAMP molecules/second/channel for Cx43, Cx46, and Cx50 channels, respectively [36]. These values were derived from the time required for the SpIH current to reach saturation in the recipient cell after delivery of cAMP to the source cell, the magnitude of gap junctional coupling between the cells, and the unitary conductance of the channels [3,36]. The last two parameters were easily measured for all three connexins, but the time to SpIH current saturation was difficult to determine for studies using Cx50 due to the very low rate of cAMP transfer. In the studies examining Cx50, the SpIH channel showed no detectable activation in half of the experiments performed and failed to achieve saturation in the other half when it was activated by cAMP permeating through Cx50 channels during the finite period from which the cell pair could be stably recorded by dual whole cell patch clamp [36]. This required approximation of the time to SpIH current saturation for Cx50, which was likely underestimated. Thus, the cAMP flux through Cx50 channels may be even lower than the calculated value of 176 molecules/second. Despite the uncertainty in the calculation of Cx50 flux, the data demonstrated that Cx43 and Cx46 channels had a permeability to cAMP that was 1–2 orders of magnitude greater than that of Cx50. The differences in cyclic nucleotide permeability between the different lens connexins were confirmed using ε-cAMP, a form of cAMP that can be directly visualized using fluorescent microscopy [40,41]. In HeLa cells expressing Cx43, ε-cAMP easily passed between cells, whereas in HeLa cells transfected with Cx50, very little transfer of ε-cAMP to neighboring cells was observed [36].

Deletion of Cx50 from the mouse lens by genetic knockout decreased epithelial mitosis during the first postnatal week and resulted in a significant reduction of lens growth [20,42,43]. These defects were not observed following knockout of either Cx43 or Cx46 in mice [44,45,46], suggesting that specific properties of Cx50 were needed for normal epithelial cell mitosis and lens growth during postnatal development. The intracellular concentration of cAMP oscillates during the cell cycle and participates in the regulation of G_0_/G_1_ transition [47,48]. Knockout of Cx50 did not alter the amount or distribution of Cx43 in lens epithelial junctions [42], and replacement of Cx50 by Cx46 by genetic knock-in resulted in the same magnitude of junctional coupling between lens epithelial cells but did not restore normal mitosis or lens growth [10,49]. These observations are consistent with the idea that the differences in cAMP permeability between connexin channels are important for regulation of lens epithelial cell division and that Cx43 and Cx46 cannot functionally replace Cx50, as they both exhibited substantially higher permeability to cAMP.

The functional activity of Cx50 has also been temporally correlated with postnatal proliferation in mouse lens epithelial cells. For example, more than 20% of epithelial cells can be labeled with BrdU on postnatal days 2 and 3, a time when Cx50 was shown to provide more than 60% of the total magnitude of epithelial coupling. After the first postnatal week, fewer than 5% of lens epithelial cells actively proliferate, and Cx50 contributes only 25% of the gap junctional coupling between them, with the majority of coupling being provided by Cx43 [20,49]. Experimental manipulation of cAMP levels altered cell proliferation in the lens epithelium [50,51,52], and the low intrinsic permeability Cx50 to cAMP could allow cells to regulate their cAMP levels independently of neighboring cells. At present, we do not know mechanistically how the loss of Cx50 leads to reduced cell division in the early postnatal lens. However, the high levels of cell-to-cell transfer of cAMP possible through Cx43 or Cx46 channels following the deletion or genetic replacement of Cx50 would be consistent with a disruption in the epithelial cells ability to autonomously regulate their cytoplasmic cAMP due to flux between neighboring cells, possibly contributing to decreased cell proliferation observed in the mouse models.

**Figure 1:**
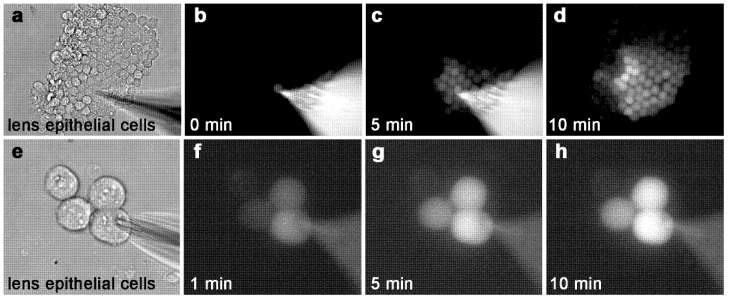
ε-cAMP passes through gap junction channels between primary lens epithelial cells. (**a**) A large cluster of explanted primary lens epithelial cells. (**b**) At time = 0 min, a patch pipette is opened, delivering ε-cAMP to one cell within the cluster. (**c**) Five minutes later, many cells within the cluster receive ε-cAMP from the single source cell. (**d**) After 10 min, more than half of the cells are labeled with ε-cAMP. In this image, the patch pipette was removed for clarity. (**e**) A group of four explanted primary lens epithelial cells. (**f**–**h**) A single wildtype epithelial cell loaded with ε-cAMP transferred the dye to two out of three neighboring cells within 10 min. Magnification a–d = 170x, e–h 730×.

We examined the ability of adult mouse wildtype epithelial cells (expressing both Cx43 and Cx50) to transfer ε-cAMP through gap junction channels in situ (Figure 1) using a primary lens epithelial explant preparation in combination with whole-cell patch clamp electrophysiology and fluorescence microscopy [46]. Six- to eight-week-old wildtype lens capsules were dissected, lightly trypsinized, and plated on glass coverslips to generate clusters of intact epithelial cells. At this age, 75% of gap junctional conductance between lens epithelial cells is mediated by Cx43 and only 25% is provided by Cx50 [49]. When the seal on a pipette that contained 5 mM ε-cAMP and was attached to a single cell within a cluster was opened, ε-cAMP transfer was easily detected into the neighboring cells with minutes. These data confirmed that ε-cAMP can permeate gap junction channels in primary wildtype lens epithelial cells in situ at a developmental age where Cx43 conductance is known to be significantly higher than that of Cx50.

### 2.2. Cx50 Has Greatly Reduced Permeability to IP_3_ Compared to CX43

The permeability of different connexins to IP_3_ was tested using a variety of experimental approaches, most often for Cx26 channels [14,53,54,55,56,57]. One frequent approach used IP_3_-mediated ER calcium release [58,59] and Ca^2+^-sensitive fluorescent dyes to detect IP_3_ permeation through connexin channels. The IP_3_ permeability of the Cx43 and Cx50 channels was compared using cell pairs loaded with the Ca^2+^ binding dye Fluo-8, where one cell was patched in whole cell mode with 500 µM IP_3_ added to the pipette solution (the donor cell). The second cell was patched in the perforated patch mode to simultaneously image cell fluorescence while monitoring gap junctional coupling (the recipient cell) [37]. All tested cell pairs expressing Cx43 showed a spike of fluorescent intensity in the recipient cell within approximately 20 s after IP_3_ was delivered to the donor cell, suggesting rapid permeation of IP_3_ through Cx43 gap junction channels. In contrast, analysis of IP_3_ permeability through Cx50 channels using the same method revealed that none of the tested cell pairs showed any spike of fluorescent intensity in the recipient cell for up to 3–4 min after IP_3_ delivery to the source cell, indicating no detectable permeation of this second messenger through Cx50 gap junction channels using this assay. The average magnitude of gap junctional conductance for the group of Cx50 cell pairs was identical to that of the Cx43 cell pairs [37]. To our knowledge, the IP_3_ permeability of Cx46 channels has not been directly tested. However, studies of how gap junctional communication mediated by different connexins can modulate calcium oscillatory behavior in cell monolayers suggest that Cx43 and Cx46 behaved differently, presumably due to intrinsic differences in IP_3_ permeability [60].

Differences in the permeability of IP_3_ through gap junction channels made from Cx43 or Cx50 could also influence lens cell proliferation and growth during development. As noted above, deletion of Cx50 decreased postnatal epithelial mitosis [20,42,43], which was not observed following knockout of Cx43 [44,46]. Ca^2+^ and IP_3_ have been shown to influence cell division synergistically [61,62,63], which has been documented in human lens epithelial cells grown in tissue culture [64]. Elevation of cytoplasmic IP_3_ levels cause Ca^2+^ to be released from the endoplasmic reticulum [65], suggesting that second messenger generation following receptor activation could be influenced by the permeability properties of connexin channels. As described above for cAMP, one can postulate that restricted IP_3_ permeability through Cx50 channels could also be important for normal postnatal epithelial cell division, if the cell’s ability to autonomously regulate IP_3_ concentration is assumed to be important. This view could help to explain why Cx43 channels, which continue to couple the epithelium in the Cx50 knockouts [42], could not compensate for loss of Cx50, as they are too highly permeable to IP_3_.

### 2.3. Cx50 Has High Permeability to Ca^2+^

Calcium ions are a third well-known soluble second messenger, and channels made from connexin proteins are both gated by and permeable to Ca^2+^ [14,66,67,68,69]. For the connexins expressed in the lens, changes in calcium concentration can markedly reduce channel conductance [70,71,72]. The Ca^2+^ permeability and gating of Cx50 was examined using transfected HeLa cell pairs loaded with Fluo-8 and a dual whole cell patch clamp, where one of the pipette solutions contained 2 mM Ca^2+^ [37]. The release of Ca^2+^ into the cytoplasm of one cell in the pair produced an increase in fluorescent intensity in the adjacent cell that reached a peak within less than 20 s, confirming rapid permeation of Ca^2+^ through Cx50 channels [37]. The conductance of Cx50 channels was also gated by Ca^2+^, as previously shown [73], but on a timescale that was significantly slower than cation permeation. It took approximately 60 s to produce a 50% decline in gap junctional conductance, and 23% of the initial junctional conductance was still present 180 s after delivery of 2 mM Ca^2+^ to the cytoplasm [37]. These studies showed that the permeation of Ca^2+^ through Cx50 channels occurred much more rapidly than the gating effect of Ca^2+^ on Cx50 conductance.

Ca^2+^ and IP_3_ permeability through gap junction channels are physiologically linked [5], as one major action of IP_3_ is to mediate Ca^2+^ release from internal stores. Cx43 showed much greater IP_3_ permeability compared to Cx50, while both connexins displayed high permeability to Ca^2+^ [37]. In addition to regulating cell division, the permeation differences of these two second messengers through lens connexin channels could also influence cataract development in the lens. Calcium contributes significantly to cataract formation [74,75], and changes in Ca^2+^ signaling in the lens epithelium may contribute to cataract progression [76]. The lens also expresses a number of G-protein coupled receptors that mediate the release of intracellular calcium through the generation of IP_3_ [77,78,79]. In addition, gap junction-mediated Ca^2+^ signaling has been observed in cultured lens epithelial cells [80]. The intercellular transfer of IP_3_ through gap junction channels is critically important for cell-to-cell propagation of Ca^2+^ signals [56,81], suggesting that the different permeability of Cx43 and Cx50 to IP_3_ could influence cataract progression. As noted above, the permeability of Cx46 channels to IP_3_ is unknown. However, Cx46 has been shown to rescue diverse forms of genetic cataract when expressed from the Cx50 gene locus [82,83,84], although the mechanism by which this occurs is unclear. If Cx46 has different intrinsic perm-selectivity to IP_3_ and/or Ca^2+^, it could help to explain this observation.

### 2.4. Summary of Large Solute Permeability between Cx43, Cx46, and Cx50

**Figure 2:**
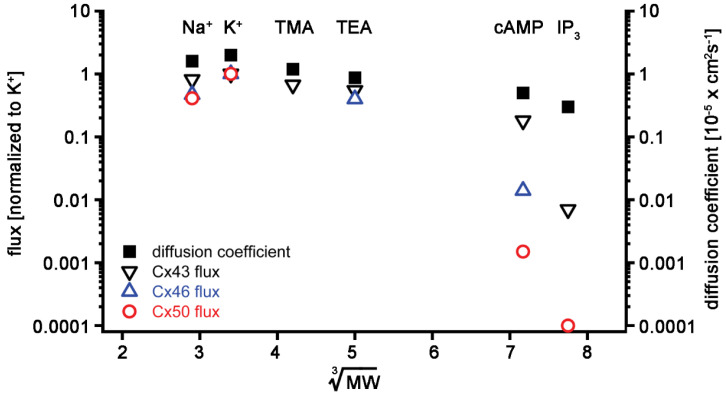
Summary of lens connexin permeability to ions (K, Na, TMA, and TEA) and second messengers (cAMP and IP_3_): flux data of different solutes were normalized to their K^+^ flux and plotted versus the cube root of their molecular weights for Cx43 (black open downward triangles), Cx46 (blue open upward triangles), and Cx50 (red open circles). The diffusion coefficients in aqueous solution were also plotted for comparison (black squares). The data were plotted on a log scale to better depict lowered flux for larger solutes like cAMP and IP_3_. See the text for references on the derivation of data.

Gap junction channels were first reported to have poor selectivity for molecules smaller than 1000 Da [85,86]; however, experiments quantitatively measuring the movement of ions and small tracer molecules between coupled cells have shown profound differences in permeation between connexin family members [33,87,88,89]. Improved technical approaches have extended this quantitative analysis of permeation to signaling molecules like cAMP and IP_3_ [3,36,37,38,53,54,57]. These studies firmly established that each connexin channel type has distinct permeability and conductance properties [3,4,5,6,38]. Figure 2 shows the flux data for Cx43, Cx46, and Cx50 to specific solutes ranging from monovalent ions to second messengers. Differences in second messenger permeability between Cx43, Cx46, and Cx50 were not well correlated with unitary conductance, as would be expected from previous studies of other connexins [87,88,89]. Data were normalized to K^+^ flux and plotted against the cube root of the solutes’ respective molecular weight. For comparison, the solutes’ diffusion coefficients (in free solution) were also plotted. Diffusion coefficients for the monovalent cations were taken from Hille [90]. For the larger solutes, values were estimated using the Stokes–Einstein equation, which considers the diffusion coefficient inversely proportional to the radius of spherical solutes [90]. If a particle has a larger radius but the same density, the volume is proportional to the cube of the radius. Therefore, the equation for diffusion becomes inversely proportional to the volume (V^3^) and thus molecular weight (MW^3^). For monovalent ions, measured unitary conductance values were converted to ion fluxes, as previously described [33,88].

All three lens connexins displayed high permeability to monovalent ions. In addition, Cx43, Cx46, and Cx50 all allowed passage of cAMP, with Cx43 being the most and Cx50 being the least permeable. IP_3_ permeability through Cx50 channels could not be detected using Ca^2+^-sensitive dyes as reporters, presumably because it was below the limit of detection of the assay. In contrast, Cx43 channels were readily permeated by IP_3_. The plotted flux values for IP_3_ represent estimates based on Cx43 and Cx50 permeation data derived from experiments using Lucifer yellow (charge −2, minor diameter 0.95 nm, and molecular mass 457 Daltons [Da]) and short oligonucleotides [33,91,92]. cAMP has a charge of −1, a minor diameter of 0.52 nm, and a molecular mass of 329 Da, whereas IP3 has a charge of −6, a minor diameter of 0.72 nm, and a molecular mass of 414 Da [55,93]. Permeability declined with increasing size for all three connexins. Size is clearly a rate-limiting factor because, if a solute’s minor diameter exceeds the pore diameter anywhere across the channel length, then that solute cannot permeate. Any solute with a minor diameter smaller than the minimum pore diameter should be permeable; however, the solute’s charge can also influence its permeation. The channel region most likely to influence solute permeability based on its charge is around the cytoplasmic opening of the pore, where access resistance in the form of fixed charges could repel or attract charged solutes [94].

Recently, the structures of native Cx46 and Cx50 have been resolved by cryo-electron microscopy at a resolution near the atomic level (approximately 3.4 Å) [95]. Although there is no equivalent atomic structure of Cx43 at this resolution, structures have been solved at 7.5 Å [96] that are in good general agreement with the higher resolution data. The availability of Cx46 and Cx50 structures will allow approaches such as comparative all-atom MD simulations to probe isoform-specific differences in perm selectivity to second messengers like cAMP and IP_3_. Cx26 channels are also permeable to both cAMP and IP_3_ [3,38,54,56]. Since an atomic level structure has also been obtained for this connexin [97], its permeability data could compliment comparative approaches in the absence of an atomic level structure of Cx43. Preliminary comparisons of the Cx26, Cx46, and Cx50 structures have already identified substantial differences at key functional sites, which could contribute to their isoform-specific permeation properties [95]. This promise of this approach is further highlighted by MD investigations of the ion permeation pathway of Cx26 and Cx30 [98].

## 3. Future Directions

The relative simplicity of the lens makes it an attractive model organ to study why epithelial tissues need to express multiple connexin subunits with distinct permeability to second messengers. The long-term hope is that insights gained about connexin diversity from the lens will be applicable to more complex epithelia such as the epidermis or respiratory epithelium [99]. The airway epithelium protects the lung from invading pathogens through a process of mucociliary clearance mediated by ciliated and mucin-secreting cells [100]. At least 10 different connexins are expressed in the airway epithelium and submucosal glands [101], and the connexin-dependent permeation of second messengers between cells may contribute to mucociliary clearance by modulating ciliary beat frequency [102]. The epidermis is the largest organ in the body and, like the airway epithelium, plays a key barrier function [103,104]. Connexins in the skin have complex expression patterns, with many cells expressing multiple subunits. Epidermal keratinocytes express several connexins, including Cx26, Cx30, Cx30.3, Cx31, and Cx43 [1,105,106]. In the epidermis, permeability of second messengers through connexin channels may play an important role in the inflammatory response to opportunistic pathogens [99]. Quantitative second messenger permeability data is currently available for approximately half of the panoply of connexins expressed in the respiratory epithelium and epidermis, so further development of these hypotheses will require more experimental data.

As the number of expressed connexin genes increases in any given tissue or organ, it becomes more difficult to ascribe connexin-specific functions that could result from differences in second messenger permeability. Despite this barrier, genetic evidence has suggested that the lack of functional redundancy that has emerged from mouse models is directly relevant to human hereditary diseases. Connexin channels are critically involved in epithelial homeostasis in humans, as illustrated by the large number of human genetic diseases resulting from connexin mutations that affect epithelial tissues [107,108]. There appears to be a lack of functional redundancy, as five connexin genes have been linked to eleven genetic skin diseases and mutations in three different connexins can cause deafness [109,110,111]. Functional studies of mutant connexins involved in epithelial disease have suggested that differences in second messenger permeability may play a role in pathogenesis. Investigation of the Cx26-V84L mutation showed that it formed functioning gap junction channels with normal permeability to monovalent ions but had markedly decreased permeability to IP_3_, suggesting that changes in second messenger permeation could underlie hereditary deafness [54]. This finding was extended to additional deafness causing mutations in both Cx26 and Cx30, strongly suggesting that connexin-mediated permeability of IP_3_ is required for normal cochlear function [57].

Multiple connexin proteins are expressed within the same organ or cell type throughout vertebrates [112] and even in the most primitive chordate animals from which connexins have been cloned [113,114]. The expression of multiple connexins within a single cell type would influence both the extent and magnitude of permeation of second messengers between adjacent cells. Understanding how variations in second messenger permeability could possibly contribute to organ development and homeostasis requires detailed knowledge derived from a variety of experimental approaches. The quantitative measurement of substantial differences in the permeation of Cx43, Cx46, and Cx50 channels to Ca^2+^, cAMP, and IP_3_ [3,36,37] is one necessary component to understanding the need for multiple connexin proteins to be expressed to maintain homeostasis in a model tissue like the lens [11,115]. A second important piece of the puzzle would be detailed knowledge of which connexins are required for what function in each specific cell type. In the case of the lens, study of genetically engineered mice have identified nonredundant functions for Cx43, Cx46, and Cx50 in both lens epithelial cells and fiber cells [10,20,45,46,49,84,116,117,118]. A third critical requirement would be detailed knowledge of channel structure to understand the molecular mechanism(s) underlying the differences in permeability to second messengers. Once again, the lens is well suited for study as atomic level structures or close approximations are available for all three of the principal connexins that it expresses [95,96]. Following decades of research effort on lens connexins, the field has currently achieved a trifecta of high-resolution atomic structures, genetic definition of critical functions, and quantitative permeability data for biologically relevant molecules. Future studies built on these foundations will undoubtedly advance our understanding of the need for connexin diversity not only in the lens but also in a wide variety of other epithelial tissues.

## Abbreviations

Cx	connexin
cAMP	3′,5′-cyclic adenosine monophosphate
IP_3_	inositol 1,4,5-trisphosphate

## References

[1] Mese G., Richard G., White T.W. (2007). Gap junctions: Basic structure and function. J Invest. Dermatol..

[2] Veenstra R.D. (1996). Size and selectivity of gap junction channels formed from different connexins. J. Bioenerg. Biomembr..

[3] Kanaporis G., Mese G., Valiuniene L., White T.W., Brink P.R., Valiunas V. (2008). Gap junction channels exhibit connexin-specific permeability to cyclic nucleotides. J. Gen. Physiol..

[4] Bukauskas F.F., Verselis V.K. (2004). Gap junction channel gating. Biochim. Biophys. Acta..

[5] Harris A.L. (2007). Connexin channel permeability to cytoplasmic molecules. Prog. Biophys. Mol. Biol..

[6] Harris A.L. (2001). Emerging issues of connexin channels: Biophysics fills the gap. Q. Rev. Biophys..

[7] Winterhager E., Pielensticker N., Freyer J., Ghanem A., Schrickel J.W., Kim J.S., Behr R., Grummer R., Maass K., Urschel S. (2007). Replacement of connexin43 by connexin26 in transgenic mice leads to dysfunctional reproductive organs and slowed ventricular conduction in the heart. BMC. Dev. Biol..

[8] Frank M., Eiberger B., Janssen-Bienhold U., de Sevilla Muller L.P., Tjarks A., Kim J.S., Maschke S., Dobrowolski R., Sasse P., Weiler R. (2010). Neuronal connexin-36 can functionally replace connexin-45 in mouse retina but not in the developing heart. J. Cell. Sci..

[9] Plum A., Hallas G., Magin T., Dombrowski F., Hagendorff A., Schumacher B., Wolpert C., Kim J., Lamers W.H., Evert M. (2000). Unique and shared functions of different connexins in mice. Curr. Biol..

[10] White T.W. (2002). Unique and redundant connexin contributions to lens development. Science.

[11] White T.W. (2003). Nonredundant gap junction functions. News Physiol. Sci..

[12] Lawrence T.S., Beers W.H., Gilula N.B. (1978). Transmission of hormonal stimulation by cell-to-cell communication. Nature.

[13] Zhang J.T., Nicholson B.J. (1989). Sequence and tissue distribution of a second protein of hepatic gap junctions, Cx26, as deduced from its cDNA. J. Cell Biol..

[14] Saez J.C., Connor J.A., Spray D.C., Bennett M.V. (1989). Hepatocyte gap junctions are permeable to the second messenger, inositol 1,4,5-trisphosphate, and to calcium ions. Proc. Natl. Acad. Sci. USA.

[15] Chaffee B.R., Hoang T.V., Leonard M.R., Bruney D.G., Wagner B.D., Dowd J.R., Leone G., Ostrowski M.C., Robinson M.L. (2016). FGFR and PTEN signaling interact during lens development to regulate cell survival. Dev. Biol..

[16] Lovicu F.J. (2006). Cell signaling in lens development. Semin. Cell Dev. Biol..

[17] Robinson M.L. (2006). An essential role for FGF receptor signaling in lens development. Semin. Cell Dev. Biol..

[18] Boswell B.A., Lein P.J., Musil L.S. (2008). Cross-talk between fibroblast growth factor and bone morphogenetic proteins regulates gap junction-mediated intercellular communication in lens cells. Mol. Biol. Cell.

[19] Le A.C., Musil L.S. (2001). A novel role for FGF and extracellular signal-regulated kinase in gap junction-mediated intercellular communication in the lens. J. Cell Biol..

[20] Sellitto C., Li L., White T.W. (2004). Connexin50 is essential for normal postnatal lens cell proliferation. Invest. Ophthalmol. Vis. Sci..

[21] Sellitto C., Li L., Vaghefi E., Donaldson P.J., Lin R.Z., White T.W. (2016). The Phosphoinosotide 3-Kinase Catalytic Subunit p110alpha is Required for Normal Lens Growth. Invest. Ophthalmol. Vis. Sci..

[22] Berthoud V.M., Ngezahayo A. (2017). Focus on lens connexins. BMC Cell Biol.

[23] Gerido D.A., White T.W. (2004). Connexin disorders of the ear, skin, and lens. Biochim. Biophys. Acta..

[24] Mathias R.T., White T.W., Gong X. (2010). Lens gap junctions in growth, differentiation, and homeostasis. Physiol. Rev..

[25] Berthoud V.M., Minogue P.J., Osmolak P., Snabb J.I., Beyer E.C. (2014). Roles and regulation of lens epithelial cell connexins. FEBS. Lett..

[26] Kar R., Batra N., Riquelme M.A., Jiang J.X. (2012). Biological role of connexin intercellular channels and hemichannels. Arch. Biochem. Biophys..

[27] White T.W., Bruzzone R. (2000). Intercellular communication in the eye: Clarifying the need for connexin diversity. Brain Res. Brain Res. Rev..

[28] Jiang J.X. (2010). Gap junctions or hemichannel-dependent and independent roles of connexins in cataractogenesis and lens development. Curr. Mol. Med..

[29] Ebihara L., Steiner E. (1993). Properties of a nonjunctional current expressed from a rat connexin46 cDNA in Xenopus oocytes. J. Gen. Physiol..

[30] Trexler E.B., Bennett M.V., Bargiello T.A., Verselis V.K. (1996). Voltage gating and permeation in a gap junction hemichannel. Proc. Natl. Acad. Sc.i USA.

[31] Srinivas M., Costa M., Gao Y., Fort A., Fishman G.I., Spray D.C. (1999). Voltage dependence of macroscopic and unitary currents of gap junction channels formed by mouse connexin50 expressed in rat neuroblastoma cells. J. Physiol..

[32] Srinivas M., Kronengold J., Bukauskas F.F., Bargiello T.A., Verselis V.K. (2005). Correlative studies of gating in Cx46 and Cx50 hemichannels and gap junction channels. Biophys. J..

[33] Valiunas V., Beyer E.C., Brink P.R. (2002). Cardiac gap junction channels show quantitative differences in selectivity. Circ. Res..

[34] Verselis V.K., Trelles M.P., Rubinos C., Bargiello T.A., Srinivas M. (2009). Loop gating of connexin hemichannels involves movement of pore-lining residues in the first extracellular loop domain. J. Biol. Chem..

[35] White T.W., Bruzzone R., Wolfram S., Paul D.L., Goodenough D.A. (1994). Selective interactions among the multiple connexin proteins expressed in the vertebrate lens: The second extracellular domain is a determinant of compatibility between connexins. J. Cell Biol..

[36] Valiunas V., Brink P.R., White T.W. (2019). Lens Connexin Channels Have Differential Permeability to the Second Messenger cAMP. Invest. Ophthalmol. Vis. Sci..

[37] Valiunas V., White T.W. (2020). Connexin43 and connexin50 channels exhibit different permeability to the second messenger inositol triphosphate. Sci. Rep..

[38] Mese G., Valiunas V., Brink P.R., White T.W. (2008). Connexin26 deafness associated mutations show altered permeability to large cationic molecules. Am. J. Physiol. Cell Physiol..

[39] Bedner P., Niessen H., Odermatt B., Willecke K., Harz H. (2003). A method to determine the relative cAMP permeability of connexin channels. Exp. Cell Res..

[40] Secrist III J.A., Barrio J.R., Leonard N.J., Weber G. (1972). Fluorescent modification of adenosine-containing coenzymes. Biological activities and spectroscopic properties. Biochemistry.

[41] Secrist III J.A., Barrio J.R., Leonard N.J., Villar-Palasi C., Gilman A.G. (1972). Fluorescent modification of adenosine 3′,5′-monophosphate: Spectroscopic properties and activity in enzyme systems. Science.

[42] White T.W., Goodenough D.A., Paul D.L. (1998). Targeted ablation of connexin50 in mice results in microphthalmia and zonular pulverulent cataracts. J. Cell Biol..

[43] Sikic H., Shi Y., Lubura S., Bassnett S. (2015). A stochastic model of eye lens growth. J. Theor. Biol..

[44] White T.W., Sellitto C., Paul D.L., Goodenough D.A. (2001). Prenatal lens development in connexin43 and connexin50 double knockout mice. Invest. Ophthalmol. Vis. Sci..

[45] Gong X., Li E., Klier G., Huang Q., Wu Y., Lei H., Kumar N.M., Horwitz J., Gilula N.B. (1997). Disruption of alpha3 connexin gene leads to proteolysis and cataractogenesis in mice. Cell.

[46] DeRosa A.M., Mese G., Li L., Sellitto C., Brink P.R., Gong X., White T.W. (2009). The cataract causing Cx50-S50P mutant inhibits Cx43 and intercellular communication in the lens epithelium. Exp. Cell Res..

[47] Abell C.W., Monahan T.M. (1973). The role of adenosine 3′,5′-cyclic monophosphate in the regulation of mammalian cell division. J. Cell Biol..

[48] Friedman D.L. (1976). Role of cyclic nucleotides in cell growth and differentiation. Physiol. Rev..

[49] White T.W., Gao Y., Li L., Sellitto C., Srinivas M. (2007). Optimal lens epithelial cell proliferation is dependent on the connexin isoform providing gap junctional coupling. Invest. Ophthalmol. Vis. Sci..

[50] Von Sallmann L., Grimes P. (1974). Effects of isoproterenol and cyclic AMP derivatives on cell division in cultured rat lenses. Invest. Ophthalmol..

[51] Grimes P., Von Sallmann L. (1972). Possible cyclic adenosine monophosphate mediation in isoproterenol-induced suppression of cell division in rat lens epithelium. Invest. Ophthalmol..

[52] Ireland M.E., Tran K., Mrock L. (1993). Beta-adrenergic mechanisms affect cell division and differentiation in cultured chick lens epithelial cells. Exp. Eye Res..

[53] Ayad W.A., Locke D., Koreen I.V., Harris A.L. (2006). Heteromeric, but not homomeric, connexin channels are selectively permeable to inositol phosphates. J. Biol. Chem..

[54] Beltramello M., Piazza V., Bukauskas F.F., Pozzan T., Mammano F. (2005). Impaired permeability to Ins(1,4,5)P3 in a mutant connexin underlies recessive hereditary deafness. Nat. Cell Biol..

[55] Hernandez V.H., Bortolozzi M., Pertegato V., Beltramello M., Giarin M., Zaccolo M., Pantano S., Mammano F. (2007). Unitary permeability of gap junction channels to second messengers measured by FRET microscopy. Nat. Methods.

[56] Niessen H., Harz H., Bedner P., Kramer K., Willecke K. (2000). Selective permeability of different connexin channels to the second messenger inositol 1,4,5-trisphosphate. J. Cell Sci..

[57] Zhang Y., Tang W., Ahmad S., Sipp J.A., Chen P., Lin X. (2005). Gap junction-mediated intercellular biochemical coupling in cochlear supporting cells is required for normal cochlear functions. Proc. Natl. Acad. Sci. USA.

[58] Gill D.L., Ghosh T.K., Mullaney J.M. (1989). Calcium signalling mechanisms in endoplasmic reticulum activated by inositol 1,4,5-trisphosphate and GTP. Cell Calcium..

[59] Berridge M.J. (2009). Inositol trisphosphate and calcium signalling mechanisms. Biochim. Biophys. Acta..

[60] Lin G.C., Rurangirwa J.K., Koval M., Steinberg T.H. (2004). Gap junctional communication modulates agonist-induced calcium oscillations in transfected HeLa cells. J. Cell Sci..

[61] Means A.R. (1994). Calcium, calmodulin and cell cycle regulation. FEBS. Lett..

[62] Lu K.P., Means A.R. (1993). Regulation of the cell cycle by calcium and calmodulin. Endocr. Rev..

[63] Whitaker M., Patel R. (1990). Calcium and cell cycle control. Development.

[64] Wang L., Wormstone I.M., Reddan J.R., Duncan G. (2005). Growth factor receptor signalling in human lens cells: Role of the calcium store. Exp. Eye Res..

[65] Berridge M.J., Lipp P., Bootman M.D. (2000). The versatility and universality of calcium signalling. Nat. Rev. Mol. Cell Biol..

[66] De Mello W.C. (1975). Effect of intracellular injection of calcium and strontium on cell communication in heart. J. Physiol..

[67] Spray D.C., Stern J.H., Harris A.L., Bennett M.V. (1982). Gap junctional conductance: Comparison of sensitivities to H and Ca ions. Proc. Natl. Acad. Sci. USA.

[68] Rose B., Loewenstein W.R. (1975). Permeability of cell junction depends on local cytoplasmic calcium activity. Nature.

[69] Peracchia C. (2020). Calmodulin-Mediated Regulation of Gap Junction Channels. Int. J. Mol. Sci..

[70] Crow J.M., Atkinson M.M., Johnson R.G. (1994). Micromolar levels of intracellular calcium reduce gap junctional permeability in lens cultures. Invest. Ophthalmol. Vis. Sci..

[71] Lurtz M.M., Louis C.F. (2007). Intracellular calcium regulation of connexin43. Am. J. Physiol. Cell Physiol..

[72] Verselis V.K., Srinivas M. (2008). Divalent cations regulate connexin hemichannels by modulating intrinsic voltage-dependent gating. J. Gen. Physiol..

[73] Chen Y., Zhou Y., Lin X., Wong H.C., Xu Q., Jiang J., Wang S., Lurtz M.M., Louis C.F., Veenstra R.D. (2011). Molecular interaction and functional regulation of connexin50 gap junctions by calmodulin. Biochem. J..

[74] Duncan G., Jacob T.J. (1984). Calcium and the physiology of cataract. Ciba. Found. Symp..

[75] Truscott R.J., Marcantonio J.M., Tomlinson J., Duncan G. (1990). Calcium-induced opacification and proteolysis in the intact rat lens. Invest. Ophthalmol. Vis. Sci..

[76] Gosak M., Markovic R., Fajmut A., Marhl M., Hawlina M., Andjelic S. (2015). The Analysis of Intracellular and Intercellular Calcium Signaling in Human Anterior Lens Capsule Epithelial Cells with Regard to Different Types and Stages of the Cataract. PLoS ONE.

[77] Duncan G., Wormstone I.M. (1999). Calcium cell signalling and cataract: Role of the endoplasmic reticulum. Eye (Lond.).

[78] Rhodes J.D., Sanderson J. (2009). The mechanisms of calcium homeostasis and signalling in the lens. Exp. Eye Res..

[79] Vivekanandan S., Lou M.F. (1989). Evidence for the presence of phosphoinositide cycle and its involvement in cellular signal transduction in the rabbit lens. Curr. Eye Res..

[80] Churchill G.C., Atkinson M.M., Louis C.F. (1996). Mechanical stimulation initiates cell-to-cell calcium signaling in ovine lens epithelial cells. J. Cell Sci..

[81] Sanderson M.J., Charles A.C., Boitano S., Dirksen E.R. (1994). Mechanisms and function of intercellular calcium signaling. Mol. Cell Endocrinol..

[82] Xia C.H., Cheung D., DeRosa A.M., Chang B., Lo W.K., White T.W., Gong X. (2006). Knock-in of alpha3 connexin prevents severe cataracts caused by an alpha8 point mutation. J. Cell Sci..

[83] Li L., Cheng C., Xia C.H., White T.W., Fletcher D.A., Gong X. (2010). Connexin mediated cataract prevention in mice. PLoS ONE.

[84] Martinez-Wittinghan F.J., Sellitto C., Li L., Gong X., Brink P.R., Mathias R.T., White T.W. (2003). Dominant cataracts result from incongruous mixing of wild-type lens connexins. J. Cell Biol..

[85] Goodenough D.A. (1978). Gap junction dynamics and intercellular communication. Pharmacol. Rev..

[86] Gilula N.B., Reeves O.R., Steinbach A. (1972). Metabolic coupling, ionic coupling and cell contacts. Nature.

[87] Goldberg G.S., Valiunas V., Brink P.R. (2004). Selective permeability of gap junction channels. Biochim. Biophys. Acta..

[88] Kanaporis G., Brink P.R., Valiunas V. (2011). Gap junction permeability: Selectivity for anionic and cationic probes. Am. J. Physiol. Cell Physiol..

[89] Veenstra R.D., Wang H.Z., Beblo D.A., Chilton M.G., Harris A.L., Beyer E.C., Brink P.R. (1995). Selectivity of connexin-specific gap junctions does not correlate with channel conductance. Circ. Res..

[90] Hille B. (1992). Ionic channels of excitable membranes.

[91] Valiunas V., Polosina Y.Y., Miller H., Potapova I.A., Valiuniene L., Doronin S., Mathias R.T., Robinson R.B., Rosen M.R., Cohen I.S. (2005). Connexin-specific cell-to-cell transfer of short interfering RNA by gap junctions. J. Physiol..

[92] Valiunas V., Wang H.Z., Li L., Gordon C., Valiuniene L., Cohen I.S., Brink P.R. (2015). A comparison of two cellular delivery mechanisms for small interfering RNA. Physiol. Rep..

[93] Valiunas V., Cohen I.S., Brink P.R. (2018). Defining the factors that affect solute permeation of gap junction channels. Biochim. Biophys. Acta. Biomembr..

[94] Banach K., Ramanan S.V., Brink P.R. (2000). The influence of surface charges on the conductance of the human connexin37 gap junction channel. Biophys. J..

[95] Myers J.B., Haddad B.G., O’Neill S.E., Chorev D.S., Yoshioka C.C., Robinson C.V., Zuckerman D.M., Reichow S.L. (2018). Structure of native lens connexin 46/50 intercellular channels by cryo-EM. Nature.

[96] Unger V.M., Kumar N.M., Gilula N.B., Yeager M. (1999). Expression, two-dimensional crystallization, and electron cryo-crystallography of recombinant gap junction membrane channels. J. Struct. Biol..

[97] Maeda S., Nakagawa S., Suga M., Yamashita E., Oshima A., Fujiyoshi Y., Tsukihara T. (2009). Structure of the connexin 26 gap junction channel at 3.5 A resolution. Nature.

[98] Zonta F., Polles G., Zanotti G., Mammano F. (2012). Permeation pathway of homomeric connexin 26 and connexin 30 channels investigated by molecular dynamics. J. Biomol. Struct. Dyn..

[99] Chanson M., Watanabe M., O’Shaughnessy E.M., Zoso A., Martin P.E. (2018). Connexin Communication Compartments and Wound Repair in Epithelial Tissue. Int J. Mol. Sci.

[100] Whitsett J.A., Alenghat T. (2015). Respiratory epithelial cells orchestrate pulmonary innate immunity. Nat. Immunol..

[101] Losa D., Chanson M. (2015). The lung communication network. Cell Mol. Life Sci..

[102] Scheckenbach K.E., Crespin S., Kwak B.R., Chanson M. (2011). Connexin channel-dependent signaling pathways in inflammation. J. Vasc. Res..

[103] Proksch E., Brandner J.M., Jensen J.M. (2008). The skin: An indispensable barrier. Exp. Dermatol..

[104] Martin P.E., Easton J.A., Hodgins M.B., Wright C.S. (2014). Connexins: Sensors of epidermal integrity that are therapeutic targets. FEBS. Lett..

[105] Kretz M., Euwens C., Hombach S., Eckardt D., Teubner B., Traub O., Willecke K., Ott T. (2003). Altered connexin expression and wound healing in the epidermis of connexin-deficient mice. J. Cell Sci..

[106] Wiszniewski L., Limat A., Saurat J.H., Meda P., Salomon D. (2000). Differential expression of connexins during stratification of human keratinocytes. J. Invest. Dermatol..

[107] Delmar M., Laird D.W., Naus C.C., Nielsen M.S., Verselis V.K., White T.W. (2018). Connexins and Disease. Cold Spring Harb. Perspect. Biol..

[108] Pfenniger A., Wohlwend A., Kwak B.R. (2011). Mutations in connexin genes and disease. Eur. J. Clin. Invest..

[109] Avshalumova L., Fabrikant J., Koriakos A. (2014). Overview of skin diseases linked to connexin gene mutations. Int. J. Dermatol..

[110] Lee J.R., White T.W. (2009). Connexin-26 mutations in deafness and skin disease. Expert Rev. Mol. Med..

[111] Lilly E., Sellitto C., Milstone L.M., White T.W. (2016). Connexin channels in congenital skin disorders. Semin. Cell Dev. Biol..

[112] White T.W., Paul D.L. (1999). Genetic diseases and gene knockouts reveal diverse connexin functions. Annu. Rev. Physiol..

[113] White T.W., Wang H., Mui R., Litteral J., Brink P.R. (2004). Cloning and functional expression of invertebrate connexins from Halocynthia pyriformis. FEBS. Lett..

[114] Sasakura Y., Shoguchi E., Takatori N., Wada S., Meinertzhagen I.A., Satou Y., Satoh N. (2003). A genomewide survey of developmentally relevant genes in Ciona intestinalis. X. Genes for cell junctions and extracellular matrix. Dev. Genes Evol..

[115] Bruzzone R., White T.W., Paul D.L. (1996). Connections with connexins: The molecular basis of direct intercellular signaling. Eur. J. Biochem..

[116] Baldo G.J., Gong X., Martinez-Wittinghan F.J., Kumar N.M., Gilula N.B., Mathias R.T. (2001). Gap junctional coupling in lenses from alpha(8) connexin knockout mice. J. Gen. Physiol..

[117] Cheng C., Xia C.H., Li L., White T.W., Niimi J., Gong X. (2008). Gap junction communication influences intercellular protein distribution in the lens. Exp. Eye Res..

[118] Rong P., Wang X., Niesman I., Wu Y., Benedetti L.E., Dunia I., Levy E., Gong X. (2002). Disruption of Gja8 (alpha8 connexin) in mice leads to microphthalmia associated with retardation of lens growth and lens fiber maturation. Development.

